# Unevenly distributed: a systematic review of the health literature about socioeconomic inequalities in adult obesity in the United Kingdom

**DOI:** 10.1186/1471-2458-12-18

**Published:** 2012-01-09

**Authors:** Abdulrahman M El-Sayed, Peter Scarborough, Sandro Galea

**Affiliations:** 1 British Heart Foundation Health Promotion Research Group, Department of Public Health, University of Oxford, Richards Building Old Road Campus Headington, Oxford, UK OX3 7LF; 2 Department of Epidemiology, Columbia University, New York, NY, USA; 3 College of Physicians and Surgeons, Columbia University, New York, NY, USA

**Keywords:** Adiposity, Overweight, Socioeconomic position, Socioeconomic status, Social class, Education, Income, Disparities, Deprivation, England, Wales, Scotland, Northern Ireland

## Abstract

**Background:**

There is a growing literature documenting socioeconomic inequalities in obesity risk among adults in the UK, with poorer groups suffering higher risk.

**Methods:**

In this systematic review, we summarize and appraise the extant peer-reviewed literature about socioeconomic inequalities in adult obesity risk in the UK published between 1980 and 2010. Only studies featuring empirical assessments of relations between socioeconomic indicators and measures of obesity among adults in the UK were included.

**Results:**

A total of 35 articles met inclusion criteria, and were reviewed here.

**Conclusion:**

Socioeconomic indicators of low socioeconomic position (SEP), including occupational social class of the head-of-household at birth and during childhood, earlier adulthood occupational social class, contemporaneous occupational social class, educational attainment, and area-level deprivation were generally inversely associated with adult obesity risk in the UK. Measures of SEP were more predictive of obesity among women than among men. We outline important methodological limitations to the literature and recommend avenues for future research.

## Background

The obesity epidemic is progressing in the United Kingdom (UK) [1,2]. Forecasting obesity prevalence among the general population in the UK from current trends, the Foresight Obesity project suggested that 60% of men and 50% of women will be obese by 2050. The findings also suggest that social class differences in obesity by socioeconomic position (SEP) may widen with time [1].

Obesity is a central contributor to cardiovascular disease, being associated with hypertension, hypercholesterolemia, and coronary heart disease [3]. Obesity is also a predictor of several other diseases of population health importance [4,5], including diabetes mellitus [6], cancer [7-9], stroke [10], and depression [11], among others [4]. Decreased life expectancy and excess mortality have also been demonstrated at both extremes of body mass index (BMI) [12,13].

Low SEP is a well-documented determinant of poor health among diverse populations. There is a large literature assessing socioeconomic inequalities in several health indicators in the UK, including socioeconomic differences in heart disease, chronic bronchitis, smoking, diet, exercise, self-rated health, and overall mortality [14,15] between rich and poor. A recently published comprehensive review about health inequalities in England highlighted SEP inequalities in morbidity, self-reported health, psychopathology, accidental injury, and mortality [16]. Several studies have also suggested that health inequalities by SEP may be widening in the UK, including life expectancy and mortality rates between the early 1980s and 2000s [16,17].

Of particular importance here is the well-documented inverse relation between SEP and obesity risk--several studies have suggested socioeconomic disparities in adult obesity in the UK, with the poor at higher risk [18-22]. A recent data briefing from the UK's National Obesity Observatory demonstrated consistent inequalities in obesity over the last decade by occupational social class (OSC) among adults, with evidence of increasing disparities among both men and women [2].

Although there have been several published reviews about the relation between socioeconomic status and obesity risk that have included UK data [23,24], to our knowledge, there has been no attempt to systematically appraise or synthesize the literature specific to this context. Our review was limited to the UK for several reasons: First, we were interested in understanding mechanisms that underpin SEP inequalities in the UK. As national health systems may influence access to health services, and may also determine the focus placed on prevention within countries, generalizing across countries may not be sensible. Second, there is a correlation between ethnicity and SEP in high-income countries, and members of ethnic minority groups have been shown to have differential risk for obesity than whites [25-29]. Countries with differing ethnic minority populations may therefore feature different relations between SEP and obesity, precluding generalization across countries. A nationally-focused review about socioeconomic differences in obesity risk in the UK is therefore warranted, as international reviews may lack the focus necessary to draw UK-specific inference that is useful for policy purposes or to direct further research to better understand the contribution of local context.

In this systematic review, we assessed the extant peer-reviewed literature published in the past 30 years about socioeconomic disparities in adult obesity in the UK. Summarizing important differences in the prevalence and determinants of obesity by different indicators of SEP in the UK, we attempted to isolate key indicators of socioeconomic position that may influence obesity risk. Moreover, we address generalizable themes and explore methodological limitations to the available literature.

## Methods

**Figure 1:**
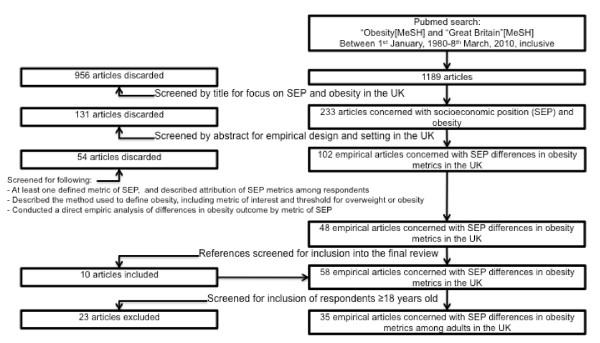
**Systematic review search strategy: Socioeconomic inequalities in adult obesity in the UK, 1980-March, 2010**.

We reviewed the peer-reviewed literature published between 1st January, 1980 and 8th March, 2010. Our review was limited to this period so as to reflect current thinking regarding the relation between SEP and health. We identified the literature reviewed through the MEDLINE database using the "http://pubmed.gov" interface. MeSH search terms "Obesity" and "Great Britain" were used to search for English-language articles published in the peer-reviewed literature. The MeSH term "Great Britain" includes any papers indexed with the following tags: "England", "Scotland", "Wales", "Northern Ireland", and "United Kingdom". All queries were carried out by the primary author during the month of March, 2010. A flow chart reporting studies excluded at each stage in the review process is shown in Figure 1.

To be included in the review, studies had to show evidence of having done each of the following:

• Considered differences in outcomes (e.g., obesity prevalence, mean BMI, etc.) by at least one defined measure of SEP, and described attribution of SEP measures among respondents

• Described the method used to define obesity, including metric of interest, and threshold for obesity utilized in analysis

• Conducted a direct empiric analysis of differences in obesity outcome by measure of SEP

The primary author extracted the following information from each paper: definition of obesity; socioeconomic position measure(s); population and setting; sample and methods; covariates included in final models; and findings and conclusions. We organized our findings by study design, considering first longitudinal analyses of the relation between SEP indicators in earlier life and obesity risk at a later point during the life course, and then moving to cross-sectional analyses of the relation between SEP indicators and obesity.

Within each study design, we first considered the relationship between individual and family-level measures of SEP and obesity. As markers of SEP at the individual or family levels, studies employed the following measures among individuals or heads of households: occupational social class (a measure of social class by employment type), educational attainment, salary scale, income, receipt of government aid, access to various resources, and/or employment history, among others. We also considered studies about the relationship between area-level measures of SEP and obesity. To assess area-level SEP, studies employed the following measures: various deprivation indices, proportions of the population by context in manual occupations, and/or proportions of the population by context renting housing from the local authority, among others. The outcomes considered in these studies included: BMI, BMI cutoffs for overweight or obesity (cutoffs employed varied by study), waist/hip ratio, waist circumference, and/or weight/height ratio, among others.

Summary measures considered included differences in mean BMI, mean waist/hip ratio, or mean waist circumference, and/or risk ratios or odds ratios of overweight or obesity (employing BMI, waist/hip ratio, or waist circumference cutoffs), among others. Given the heterogeneity in area-level and individual/household-level measures of SEP employed, as well as the multiplicity of metrics of obesity in the literature reviewed here, a meta-analysis of the results was not pursued.

## Results

Additional file 1: Table S1 features a detailed review (including SEP indicator used, definition of obesity, setting and population, sample and methods, covariates included in final models, and findings and conclusions) of each study. Our original search yielded 1189 articles, 233 of which were judged to consider the relation between SEP and obesity in the UK after screening by title. Upon screening by abstract for empirical articles set in the UK, we were left with 102 articles. After reading the remaining articles, another 54 were discarded because they did not meet the specified inclusion criteria. Reference lists from the remaining 48 articles were searched, and yielded a further 10 articles which fulfilled the inclusion criteria, leaving a total of 58 articles. Finally, 23 articles did not include outcome measure among respondents older than 18 years, and were excluded, yielding a total of 35 articles reviewed here.

Studies in this review featured two empirical designs: 20 studies were longitudinal analyses, and the remaining 15 were cross-sectional. Only four studies included socioeconomic measures collected at multiple levels (area-level, household/individual-level), and only two of these studies utilized multilevel modeling approaches. None of these studies utilized systems modeling approaches in analysis.

There were 19 studies that analyzed representative data from at least one country in the United Kingdom: 12 studies reported on data from Wales; 14 reported on data from Scotland; and 19 studies reported on data from England. There were no studies that considered data from Northern Ireland. The remaining 16 of the studies we reviewed analyzed data from regional datasets from localities throughout the UK (London, Newcastle, etc.).

### Findings from longitudinal analyses

#### Childhood socioeconomic position and obesity in adult life

Sixteen studies were concerned with head-of-household social class at birth or during childhood and risk for obesity in adulthood [18-22,30-41]. Among these studies, poor socioeconomic position in childhood was shown generally to be associated with adulthood obesity in all studies, with a few exceptions by gender or metric of obesity [18-22,32-41].

For example, using longitudinal data about 2,659 men and women from the 1946 British birth cohort, a population representative sample of infants born in one week in 1946 from England, Scotland, and Wales, Hardy and colleagues [20] found that low paternal OSC during childhood was associated with higher BMI at age 43, even after adjusting for adult social class and educational attainment. Another study followed 9,377 men and women from the 1958 British birth cohort, a similar population representative sample of infants born in one week in 1958 in England, Scotland and Wales. Similarly, this study found that low childhood paternal OSC was associated with higher mean BMI even after adjusting for adulthood social class [40]. These findings have been supported by several other longitudinal studies that have demonstrated relations between low childhood head-of-household OSC and higher risk for obesity in adult life [18,19,21,22,30-38,40,41]. Only one study found that the relation between low paternal OSC (at birth) and higher BMI was attenuated after adjustment for a potentially confounding covariate--among a cohort of 7,184 children born in Aberdeen, Scotland between 1950 and 1956, Lawlor and colleagues demonstrated that the relation between paternal OSC (at birth) and BMI no longer persisted in models adjusted for educational attainment.

Several studies reported gender differences in the relation between childhood head-of-household OSC and obesity risk [18,21,22,32,39], suggesting that paternal OSC during childhood may be a more rigorous determinant of obesity among woman than among men. A study by Hart and colleagues [21] found no relationship between childhood paternal OSC and any metric of obesity (BMI > 30 kg/m^2 or mean waist circumference) among men, but non-manual childhood paternal OSC was associated with 1.8 cm (*p *= 0.016) lower waist circumference among women. In another study, using data from both the 1946 and 1958 birth cohorts, Power and colleagues [39] found that despite an association between paternal OSC and obesity (BMI ≥ 30 kg/m^2) risk among men in the 1958 cohort, there was no relation among men from the 1946 cohort in either adjusted or unadjusted models. However, there was a relationship between paternal OSC and obesity risk among women in adjusted models in both cohorts. Moreover, odds of obesity among those with manual childhood paternal OSC were higher among women than among men. This finding was also supported by Langenburg and colleagues [32], who demonstrated a significant interaction between paternal OSC and gender, indicating that the relation between childhood paternal OSC and adult obesity risk may be stronger among woman than among men. However, it is important to note that in one separate analysis of the 1946 British birth cohort, opposite results were demonstrated: in fully-adjusted models (including adult OSC) childhood paternal OSC was not associated with mean waist-hip ratio or waist circumference among women, although there was an association among men [22].

#### Early adult socioeconomic position and obesity in later adult life

Three studies were concerned with measures of socioeconomic position in early adult life and risk for obesity in later adult life. This literature is unclear about the relation between measures of SEP in adulthood and obesity in later adult life. For example, in an analysis of the 1958 British birth cohort, Power and colleagues [38] showed that in unadjusted models, as well as those adjusted for both paternal OSC at birth and current OSC, social class at 23 was inversely associated with obesity (BMI ≥ 30 kg/m^2) at age 30 among men. Among women, social class at age 23 was inversely associated with obesity (BMI ≥ 30 kg/m^2) at age 30 in unadjusted models, as well as those adjusted for childhood paternal OSC, but the relation was attenuated once adjusted for current adult OSC. Similarly, another study analyzed data from the 1946 British birth cohort and showed that in bivariate models, lower OSC at age 26 was associated with higher waist-hip ratio, waist-height ratio, waist circumference, and BMI at age 53 among women, but only waist-hip ratio, waist-height ratio, and BMI at age 53 among men. After adjusting for childhood paternal OSC and current OSC, lower OSC at age 26 was associated with higher waist-hip ratio, waist-height ratio, and waist circumference among women, but was not associated with any outcome among men [32]. A third study of nearly 8,000 civil servants in London found that early adulthood employment grade was strongly inversely associated with obesity (BMI and waist-hip ratio) in later life [42].

#### Social mobility and obesity

Two studies considered the relation between social mobility and adult obesity. Langenburg and colleagues analyzed data from the 1946 British birth cohort and showed that among men, waist-hip ratios differed significantly between those in stable manual and stable non-manual OSCs, and that those who were either upwardly or downwardly mobile did not differ significantly from any other group, and intermediated waist-hip ratios between the groups they left, and those they entered. Similar findings were reported among women, although those in the stable non-manual OSC, as well as those who were upwardly mobile had significantly lower waist-hip ratios than those in the stable manual group [32]. Another study analyzed data about over 2,000 individuals in Renfrew and Paisley, and found that there were no significant differences in obesity (BMI ≥ 30 kg/m^2) risk among the upwardly or downwardly mobile (derived from paternal OSC and adult OSC) relative to those who were socially stable [21].

#### Socioeconomic position and trajectories in obesity

Three studies were concerned with measures of SEP and trajectories in obesity in the UK, suggesting generally that socioeconomic disparities in obesity are widening. One study analyzed data about nearly 8,000 male and female government employees in London, and found that those in the lowest employment grade had higher odds (approximately of 2.5 [men] and 2.8 [women]) of experiencing a BMI increase of greater than 6 kg/m^2 over an average of 25 years follow-up compared to those in the highest employment grade [41]. Another analysis found that although area-level deprivation (Townsend Material Deprivation Score [43]) was not associated with BMI increase among men or overall, this measure was associated with BMI increase among women after adjusting for baseline BMI, as well as among those who were obese at baseline [44]. However, one analysis of the 1958 British birth cohort found contrasting results--there was a decrease in the educational gradient in obesity between ages 23 and 33 among both men and women [45].

#### Area-level socioeconomic indicators and obesity

One study considered an area-level measure of SEP and risk for obesity. This study, by Lyratzopoulos and colleagues [44], detailed above, among nearly 20,000 men and women in Stockport, showed that the Townsend Material deprivation score [43], was associated with no significant trend in mean annual BMI increase by deprivation, either overall or among those who were not obese, although there was a significant association between deprivation and annual increase in BMI among those who were obese at baseline. Moreover, among women, after adjusting for baseline BMI, there was also a significant association between deprivation and annual increase in BMI [44].

### Findings from cross-sectional analyses

#### Occupational social class and obesity

Twenty-seven studies were concerned with the cross-sectional relations between OSC and adult obesity in the UK [18-22,30-33,35,37,40,42,46-59]. This literature suggests that low OSC is associated with higher risk for obesity, as 25 of these 27 studies found significant associations. However, several found differences in this relation by gender [18,21,32,42,57], and one found differences in the relation by ethnicity [48]. Only two found no association at all [30,46].

Among studies that found an association between OSC and obesity [18-22,31-33,35,38,40,42,47-59] was a study by Power and colleagues [53] among a sample of over 7,000 from the 1958 British birth cohort, which found that low OSC was significantly associated with higher obesity (BMI ≥ 30 kg/m^2) risk. Another study among over 30,000 respondents from the Health Surveys for England in years 2000-2003 found that OSC was associated with overweight (BMI ≥ 25 kg/m^2) across urban and rural settings in England [59]

Several studies found differences in the relation between occupational social class and obesity by gender [18,21,32,42,47,55,57]. These studies suggest that the relationship between OSC and obesity may be stronger among women than among men. For example, Brunner and colleagues, in a study of nearly 7,000 British civil servants in London, found that after adjusting for childhood paternal OSC, adult OSC was inversely associated with mean BMI, waist/hip ratio, and waist circumference among women, but only BMI and waist/hip ratio among men [18]. Another study of over 15,000 respondents to the Health Survey for England in 1996 found that OSC was only associated with obesity (BMI ≥ 30 kg/m^2) risk among women after adjusting for potential confounders. These studies are supported by several others with similar findings [21,32,47]. However, two studies showed contradictory results, finding an association between OSC and obesity among men, but not women [43,55].

One study noted differences in the relation between OSC and obesity by ethnicity. Among a multiethnic sample in Newcastle, Bhopal and colleagues [47] showed that among European White men, low head-of-household OSC (usually but not always referring to the OSC of the man in question) was associated with high waist/hip ratio, but not among Indian, Pakistanis, or Bangladeshi men. Similarly among women, low head-of-household OSC was associated with high waist circumference, waist/hip ratio, and BMI among European women, but not among any other ethnic group [47].

Two studies found no association between OSC and obesity risk [30,46]. For example, a study of nearly 5,000 men and women between 45 and 59 in Caerphilly and Bristol found no association between OSC and body mass index, although there was a non-significant tendency toward lower BMI among those in higher OSCs [46].

#### Education and obesity

There were four studies concerned with the relation between education and obesity in the UK. In general, low education was associated with higher risk for obesity. One study detailed above [33], found that education explained the relation between OSC and obesity among a cohort of 7,000 adults born in Aberdeen between 1950 and 1956. Among just over 15,000 respondents to the 1996 Health Survey for England, Wardle and colleagues [57] found that age of the mother at time of completing education was inversely associated with obesity (BMI ≥ 30 kg/m). Bhopal and colleagues found that the association between education and obesity may differ by ethnicity--they found that low education was associated with high waist/hip ratios among Indian women, but not among other groups. One study found no association between education and obesity--Gulliford studied parents of 5,229 children who entered the National Study of Health and Growth between 1973 and 1976 and 1982-88 and found that relations between education and BMI dissipated after adjustment for OSC [51].

#### Other individual and household socioeconomic measures and obesity

Several studies considered relations between other individual and household-level measures of SEP, including rented vs. owned accommodations, access to a car, and government financial aid, and obesity. Rona and Morris [53] studied over 7,000 parents aged 20-55 in England and Scotland, and showed that head-of-household unemployment was associated with higher weight for height among men and women in adjusted models in England, as well as men in Scotland. Riva and colleagues [59] found that access to a car in cities other than London was associated with overweight (BMI ≥ 25 kg/m^2) among 30,000 respondents to the Health Surveys for England, 2000-2003. However, there was no association between years of residence in local area and risk of overweight in this study [59]. Wardle and colleagues [57] analyzed data from the 1996 Health Survey for England and found that receiving government aid was associated with higher odds of obesity (BMI ≥ 30 kg/m^2) in adjusted models among both men and women, and that living in rented vs. owned accommodation was associated with higher odds of obesity in adjusted models among women, but not men.

#### Area-level deprivation and obesity

Six studies considered relations between area-level measures of deprivation and obesity in the UK, with mixed findings. For example, a study by Ellaway and colleagues [49] found that neighborhood poverty was associated with higher mean BMI and waist circumference among nearly 700 adults aged 40 and 60 in the West of Scotland. Another study found no significant relations between the Jarman Underprivileged area score or average annual unemployment by electoral wards and obesity (BMI ≥ 30 kg/m^2) among 3,877 adults in the Rotherham Health authority [52]. A third study found that town-level proportion of manual workers was associated with obesity (BMI ≥ 28 kg/m^2) among 7,735 men aged 40-59 in the British Regional Heart Study [58].

Two particularly powerful studies used data about SEP measures at multiple levels and multilevel modeling techniques to analyze relations between area-level measures of SEP and obesity in the UK. Among 30,000 respondents to the Health Surveys for England, 2000-2003, Riva and colleagues found that area-level deprivation was associated with risk of overweight in English cities other than London, as well as in semi-rural villages, even after adjusting for OSC [59]. Moon and colleagues explored urban-rural differences in socioeconomic predictors of obesity among 18,526 respondents to the 1998-1999 Health Surveys for England [56]. Using several predictors at the Ward level, including average annual income, male economic inactivity, proportion with low and high social grades, and proportion renting from the local authority, and adjusting for individual-level SEP indicators, they found that ward-level proportion with low social grade was associated with higher obesity (BMI ≥ 30 kg/m^2) risk, ward-level proportion renting from the local authority was associated with higher risk for both overweight (BMI ≥ 25 kg/m^2) and obesity, and that ward-level proportion with high social grade was protective against overweight [56].

## Discussion

A systematic review of the peer-reviewed literature about socioeconomic inequalities in adult obesity in the UK published between 1980 and 2010 found that socioeconomic indicators of low SEP throughout the life course as well as in cross-sectional analyses, including head-of-household OSC at birth and during childhood, earlier adulthood OSC, current OSC, educational attainment, and area-level deprivation were reliably associated with higher obesity risk in the UK. Notably, several indicators, including low head-of-household childhood OSC and low adulthood OSC, were found to be more strongly associated with obesity among women than among men. There may also be ethnic differences in the relation between SEP and obesity risk.

This is the first systematic review, of which we are aware, to consider the relation between SEP and obesity in the UK. However, our findings are supported by other systematic reviews about socioeconomic inequalities in obesity in high-income contexts that have shown an inverse relation between SEP and obesity risk [23,24,60]. Our findings are also supported by the conceptual literature about socioeconomic inequalities [61]. In their work on fundamental causes, Link and Phelan posit that higher SEP will always predict better health because SEP, through access to more knowledge, money, power, social connectedness, and prestige, affords access to resources that can optimize health across societies in all times [61]. In a society, such as the UK, where cardiovascular disease is responsible for a third of all deaths [62], it is plausible, then, that lower SEP should predict higher risk for obesity, a critical modifiable risk factor for cardiovascular disease.

There are several mechanisms that may mediate the relation between SEP and obesity risk in high-income countries. First, education is a principle component of SEP, predicting both income and social class. Independently of material pathways, however, education, itself, may also predict obesity risk via access to health information and perceived agency [33]. Education portends health literacy, as less-educated individuals may lack the numeracy required to understand health advice from health providers [63] or the literacy required to access health information available in other media. The resultant lack of information among the less-educated may then shape food and physical activity choices, as has been demonstrated in findings from the Low Income Diet and Nutrition Survey, which showed that those without educational qualifications had lower fruit and vegetable consumption and higher consumption of energy-dense foods as compared with those with even the educational lowest qualifications [64]. Moreover, less educated individuals may lack the confidence or perceived agency to improve their health. For example, a recent study of 1,967 women aged 18-34 in Scania, Sweden demonstrated the constellation of low education and behaviors portraying low locus of control among overweight and obese women relative to their counterparts who were underweight or had normal weight [65].

Higher income and social class also operate to mitigate obesity risk. Most directly, income, as well as social class (highly-correlated with income) may promote a healthier diet via direct access to healthier food options [64]. These factors may also protect against obesity via more consistent access to food. Food insecurity, defined as limited or uncertain availability of nutritionally adequate and safe foods [66], may promote obesity by incentivizing binge-eating--as food insecure individuals may be uncertain about the availability of their next meals, they may binge on meals when they are, in fact, available. Aggregated over time, this compensatory behavior can increase obesity risk [67,68].

Income and social class may also shape residential decisions. Area-level SEP may predict obesity risk in important ways [44], and neighborhoods may shape obesity risk via several mechanisms [69]: Low-income neighborhoods may have less green space and lower walkability, which may discourage physical activity [69]. Furthermore, low-income neighborhoods may limit access to healthy foods, limiting the quality of diets among residents [69]. Low-income neighborhoods are also characterized by lower social capital, a measure of inter-member trust and support that is influenced by the degree of crime, safety, and disorder in a context. In that vein, a recent study by Poortinga and colleagues demonstrated that low social capital may increase obesity risk [70], suggesting that even beyond access to material resources, characteristics of communities in low-income neighborhoods may influence obesity.

The distinct social history of the cohort of UK adults considered in a large number of the studies reviewed here may also be important. The World War II and reconstruction eras, into which many of these adults were born, were turbulent economic times in the UK [71]. Between 1939 and 1955, essential food supplies, clothing, and household products were rationed by the British government to bolster the war effort and accelerate post-war reconstruction [71]. A consequence of this policy, however, was the accentuation of class differences in food access, as the wealthy were able to supplement their rations via other means [71].

Several studies have suggested that the macronutrient environment in early development may be particular important in determining obesity risk in later life [72-74]--and that food scarcity during development may predict obesity in later life [74]. Indeed, findings from many of the studies we reviewed here suggest that exposure to low socioeconomic position in childhood may increase risk for adult obesity [18-22,32-41]. As a substantial proportion of the adults sampled in the studies we reviewed here were born during the era of government food rationing, it is plausible that some of the adulthood differences in obesity risk by SEP observed here may reflect, in part, intrauterine or early childhood macronutrient scarcity, particularly among children from low SEP households who maintained low SEP in adulthood.

### Gender differences in the relation between socioeconomic position and obesity

Of the 35 articles reviewed here, 17 showed differences in the relation between SEP and obesity risk by gender [18,21,31,32,34,38-40,42-45,47,54,55,57,58]. Overwhelmingly, the literature suggests that SEP measures are more strongly and reliably associated with obesity among women than among men (as demonstrated by 13 of 17 studies) [18,21,31,32,39,40,42,44,47,54,55,57,58].

As discussed above, studies found consistent differences in both the relations between childhood OSC and risk for obesity in adulthood, as well as adulthood OSC and concurrent risk for obesity by gender. Moreover, one study found that although area-level deprivation (Townsend Material Deprivation Score [43]) was not associated with BMI increase among men or overall, it was associated with BMI increase among women [44].

The finding that SEP may be more strongly inversely associated with obesity risk among women than among men is consistent with other systematic reviews of the literature about socioeconomic inequalities in obesity in high-income contexts [24,60]. While it remains unclear why SEP may be more strongly and reliably associated with obesity risk among women, this dimorphism has a plausible explanation. Aside from one study [44], OSC was the socioeconomic measure employed in all of the other studies that found gender differences in the relation between SEP and obesity [18,21,31,32,34,38-40,42,43,45,47,54,55,57,58]. The literature about socioeconomic measures in the UK suggests that women in the same occupations, and therefore the same OSCs, may receive lower remuneration than men [75,76]. Women workers may also be concentrated into fewer and lower-paid occupations per OSC classification than men [77]. In this way, OSCs may not be comparable across genders, and lower OSC categories among women may reflect substantially more disadvantage relative to their male counterparts. This difference in SEP indicated by OSC by gender, therefore, may in part explain the stronger relationship between OSC and obesity risk among women as compared to men in this literature.

### Methodological limitations of the extant literature

While the present review draws attention to important socioeconomic gradients in obesity risk in the UK, there are several limitations to the present literature that challenge our understanding of the relation between SEP and obesity risk among adults in this context: 1) the overreliance on occupational social class (OSC) as the principle socioeconomic measure in extant studies, 2) few studies (three out of 35) that have simultaneously considered SEP indicators at both the area-level and the individual/household-level, 3) few studies (two out of 35) have utilized multilevel or systems modeling techniques to assess the potential for socioeconomic influences on obesity at multiple levels, and 4) a paucity of studies (one out of 35) that have utilized ethnically diverse datasets, and/or assessed differences in the relation between SEP indicators and obesity by ethnic group.

The first methodological limitation to the extant literature is the overreliance on occupational social class (OSC) as a measure of SEP in studies concerned with inequalities in obesity in the UK. To frame this limitation, of the studies reviewed here, only 11 out of 35 considered SEP indicators other than OSC. And among those 11 studies, there were nearly twenty other measures of SEP considered. The next most utilized SEP indicator was education, which was only considered in four (as compared to 25) studies reviewed here. Taken together, these findings suggest that our understanding of SEP inequalities in obesity in the UK is heavily dependent on the OSC indicator, and that there are relatively few comparably well-studied indicators upon which to base our understanding of SEP disparities in obesity in the UK.

The Occupational Social Class indicator was developed in 1913 by British Registrar General THC Stevenson, and has regularly been collected in UK datasets since that time [20,60,78]. As termed by Stevenson, the indicator was meant to capture "standing within the community" or "culture" [78,79]. Shown to be reliably predictive of morbidity and mortality [20,79,80], similar indicators have been adapted in several other European countries [77].

There are several deficiencies to the OSC as a measure of SEP (for review, see Krieger and colleagues [77]), because of which, the literature about SEP disparities in adult obesity in the UK is challenged by an overreliance on the indicator. First, there may be considerable heterogeneity in exposure to poverty and potentially pathogenic occupational exposures by ethnicity and gender within a given OSC [77]. For example, as noted above, women, along with ethnic minorities in the same occupations have been shown to receive lower remuneration than men and whites in the UK, even after accounting for education and work experience [77,80]. Moreover, evidence in this context has suggested that women workers may be concentrated into fewer and lower-paid occupations per OSC classification than men [79]. Second, the OSC may not accurately identify the SEP of individuals outside of the market labor force, such as the unemployed, retired adults, children, and individuals employed in informal sectors, such as homemakers [81]. Although head-of-household OSC measures may be used as proxies for measuring SEP among individuals who fall into the above classifications, these proxies do not account for differences in family structure and/or dependency in relation to the head-of-household. Third, this measure may not be comparable across economic spatial or temporal contexts, as distributions of wealth, prestige, and exposure to potentially pathogenic occupational hazards may be different across occupations in different spatial and temporal contexts. This heterogeneity may therefore limit comparisons of the relations between OSC and health metrics across contexts in space and time.

The second two limitations to our understanding of SEP inequalities in adult obesity, that only three studies that have simultaneously considered SEP indicators at both the area-level and the individual/household-level, and that only two have utilized multilevel techniques (none that have used systems modeling techniques) to assess the potential for socioeconomic influences on obesity at multiple levels in the UK, are of fundamental importance. The notion that individuals may interact, and thus be influenced by, their ecological contexts is foundational in population science research [82-85]. Studies concerned with SEP inequalities in adult obesity which only consider variation in obesity using measures of SEP at the individual or household level (29 of 35 studies reviewed) may not appropriately account for the etiologic impact of ecological poverty on obesity, and therefore may yield an incomplete assessment of the association between SEP and obesity. Rather, studies that simultaneously consider both individual and area-level factors as determinants of outcomes are most appropriate, given the following three considerations: First, individuals interact with their ecological contexts, and are therefore potentially influenced by them [82-85]. Second, area-level SEP variables may be poor proxies for individual-level SEP. And third, quantifying the direct and indirect contributions of area-level SEP indicators to outcomes of interest in epidemiologic analyses that do not include individual-level indicators is challenging. Over the past several years, therefore, epidemiologists have begun to conceptualize and analyze etiologic models of disease from a multilevel perspective [86], which has presented a movement away from traditional models focusing exclusively on indicators at the individual-level, or proxies thereof [87]. Accounting for clusters within data nested at multiple levels of aggregation, multilevel models, allow the researcher to estimate mutually-adjusted exposure effects across levels of influence [82]. This approach to etiologic conceptualization and analysis has allowed investigators to consider how characteristics at several levels of influence--individuals, households, neighborhoods, cities, countries, and societies--may produce, individually and collectively, health and disease [86].

Emerging from this paradigm, as well as responding to a need for novel approaches to epidemiologic analysis, and the limitations of deterministic modeling, complex systems approaches utilize stochastic modeling techniques, allowing researchers to capture dynamic, bi-directional, and relational interactions between "exposures" and "outcomes" at several levels of influence [86]. Therefore, these approaches may be ideal for investigating the etiology and consequences of SEP inequalities in obesity in high-income contexts, such as the UK. In the absence of collective study of SEP measures at multiple levels of influence using multilevel or complex systems tools, our understanding of SEP disparities in obesity and their etiologies remains limited.

The fourth limitation to our understanding of the relation between SEP and obesity in the UK is a paucity of studies that have of utilized ethnically diverse datasets, and/or assessed differences in the relation between SEP indicators and obesity by ethnic group. There was only one study [47] concerned with differences in the relation between SEP and obesity by ethnic group, and this study found, as discussed above, potentially important differences in the relation between SEP and obesity by ethnic group. Many longitudinal studies about SEP disparities in obesity (8 of 20) utilized data from the 1946 and 1958 British birth cohorts, which do not adequately represent ethnic minorities in the UK of the 21st century [88].

Ethnic minorities are a large and growing subpopulation in the UK. Data from the most recent UK census (2001) [89] indicates that ethnic minority groups in the UK comprise over 8% of the total population, with about 4.6 million ethnic minority individuals in the UK. There are important socioeconomic differences between the ethnic minority and white UK populations. Ethnic minorities tend to be of lower SEP than their white counterparts. For example, Pakistani and Bangladeshi groups have the lowest proportions in "managerial and professional occupations" OSCs, and Bangladeshis and Black Africans in the UK have the highest proportions of children eligible for free school meals [90]. Ethnic minorities are more likely to be unemployed, and to have no educational qualifications [91]. Disparities in the healthcare experiences of ethnic minorities and whites have also been documented. For example, ethnic minorities are less likely to report positive experiences with healthcare providers compared to whites [90,92].

Given the size of the ethnic minority population in the UK, as well as the substantial demographic differences between these populations and the general population in this context, it is plausible, as supported by the extant work [47], that there are important differences in the relation between SEP and obesity by ethnic group. The paucity of studies that have considered this relation, or have used ethnically-representative datasets presents a limitation to our understanding of inequalities in obesity, as it limits our understanding of how ethnicity and SEP may interact to determine obesity risk.

## Limitations

There are several limitations that should be considered when interpreting the findings reported here. First, because our inclusion criteria limited the studies reviewed here to those published in the peer-reviewed literature, the inferences we have drawn may be subject to a publication bias. Although we used relatively permissive inclusion criteria, and included studies analyzing many of the largest health surveys in the UK, our findings may not accurately reflect current knowledge about SEP and obesity in the UK. Second, our search strategy included a query of only one database, and therefore, it is plausible that some of the literature about the relation between SEP and obesity risk may not have been represented in our findings. However, a detailed query of the citations of all studies found via our initial search was conducted to minimize this possibility. Moreover, we were interested in reviewing the public health and medical literatures. In this light, a recent study of the utility of the four most prominent biomedical databases demonstrated that MEDLINE was the optimal tool for searches of the biomedical literature [93]. Third, there was substantial overlap with respect to the health surveys analyzed in the studies we reviewed, which may limit the breadth of our findings. However, this is a limitation imposed by the literature itself and was unavoidable. Fourth, our findings were organized by a data type, and by SEP indicator. This organizational scheme may have, in part, shaped the inferences drawn here. Fifth, our findings were limited to studies about socioeconomic disparities in obesity risk among adults in one European country. It would therefore be inappropriate to generalize our findings to other contexts.

## Conclusion

Our systematic review of the peer-reviewed literature between 1980 and 2010 demonstrated considerable inequalities in obesity by SEP in the UK. However, there remain several limitations to our understanding of the relation between SEP and obesity in the UK. Considering these limitations, we suggest that investigators interested in SEP disparities in obesity in the UK pursue three avenues of inquiry. First, future studies about the relation between individual-level SEP and obesity might operationalize individual-level SEP using common measures of SEP other than OSC, including educational attainment and/or income. Second, the conceptualization and analysis of future studies in this area should consider multilevel and complex systems approaches that account for SEP influences at multiple levels, including the individual, household, and area levels, on risk for obesity in this context. Third, future work may explicitly examine differences in the relation between SEP and obesity by ethnicity in the UK, as current work has suggested that SEP may interact with ethnicity to influence obesity in important ways.

## Abbreviations

SEP: Socioeconomic position; OSC: Occupational social class; UK: United Kingdom; BMI: Body mass index.

## Competing interests

The authors declare that they have no competing interests.

## Authors' contributions

AME conceived the review, primarily conducted the literature search and review, and drafted the manuscript. PS was involved in the literature search and review, advised on the search strategy, and critically edited the manuscript for intellectual content. SG advised on the search strategy and critically edited the manuscript for intellectual content. All authors read and approved the final manuscript.

## Pre-publication history

The pre-publication history for this paper can be accessed here:

http://www.biomedcentral.com/1471-2458/12/18/prepub

## Supplementary Material

Additional file 1 **Table S1**. Studies about socioeconomic inequalities in adult obesity in the UK, 1980-2010 [incl. [19-22,30-35,37-42,44-46,48-59,67,68,94,95]]. Click here for file
