# Supplementary material for: Unevenly distributed: a systematic review of the health literature about socioeconomic inequalities in adult obesity in the United Kingdom
Source: BMC Public Health. 2012 Jan 9;12:18. doi: 10.1186/1471-2458-12-18 (PMC3293718; doi:10.1186/1471-2458-12-18)
Supplement: Additional file 1 — Table S1. Studies about socioeconomic inequalities in adult obesity in the UK, 1980-2010 [incl. [19-22,30-35,37-42,44-46,48-59,67,68,94,95]]. [file 1471-2458-12-18-S1.DOC]

Table S1. Studies about socioeconomic inequalities in adult obesity in the UK, 1980-2010

|  |  |  |  |  |  |  |  |
| --- | --- | --- | --- | --- | --- | --- | --- |
| **Reference** | **Inequality Measure** | **Obesity-related outcome** | **Location** | **Sample** | **Methods** | **Covariates included in final models*** | **Conclusions** |
| **Longitudinal** |  |  |  |  |  |  |  |
| Blane et al., 1996 [30] | Paternal occupational social class; adult social class | Mean body mass index | West of Scotland | 5,645 males aged 35-64 in the West of Scotland collaborative study between 1970-1973 | A longitudinal analysis of the relations between past and present social circumstances and cardiovascular disease risk factors | Age, paternal social class, adult social class | Low paternal social class was significantly related to high mean body mass index, although adult social class was not. |
| Brunner et al., 1999 [48] | Salary scale grade; paternal occupational social class | BMI, waist circumference, and waist/hip ratio | London | 4,774 men and 2,206 women born between 1930-1953 who took part in the Whitehall II study | A longitudinal analysis of the relations between childhood and adulthood socioeconomic status and cardiovascular disease risk factors | Age, paternal social class, adult social class, and educational attainment | In age-adjusted analyses, paternal social class was inversely associated with BMI, but not waist/hip ratio, or waist circumference among men, and all measures among women. Adult social class was inversely associated with each outcome among both men and women. After adjusting for adult social class, father's social class was inversely associated with BMI, but not waist/hip ratio, or waist circumference among men, and all measures among women. Adult social class remained associated with male waist/hip ratio but not BMI and both outcomes among women after adjusting for adult education and paternal social class. |
| Ferrie et al., 2007 [19] | Employment grade | Overweight (BMI 25.0-29.9 kg/m^2) and obesity (BMI ≥ 30.0 kg/m^2) | Whitehall, London | 2,564 female and 5,853 male British civil servants between 35-55 on entry into the Whitehall II study | A longitudinal study of the relation between obesity and work absenteeism among British civil servants | Employment grade, age | Employment grade was associated with mean BMI among both men and women, with lower employment grades having higher mean BMI. Among men, the mean BMI among the lowest grade was 25.3 kg/m2 compared to 24.5 kg/m2 among the highest grade. Among women, the mean BMI among the lowest grade was 25.1 kg/m2 compared to 23.9 kg/m2 among the highest grade. |
| Hardy et al., 2000 [20] | Paternal occupational social class; adult occupational social class; | Body mass index | England, Wales, and Scotland | 2,659 total from a cohort of 2,548 women and 2,814 men born in one week in 1946 in England, Scotland, and Wales | Longitudinal analysis of the relation between childhood socioeconomic status and obesity in adulthood | Sex, age, sex*age, age^2, sex*age^2, relative weight at age 7, relative weight at age 11, relative weight at age 14, paternal social class, relative weight at age 14*age, sex*relative weight at age 14, education | Mean BMI increased with age among all groups. Those with manual social class during childhood had higher mean BMI and a faster rate of increase than those from non-manual social class households during childhood. At all ages, those from a manual social class had a greater proportion classified as overweight and obese than those from non-manual backgrounds. Even after adjustment for childhood social class and educational attainment, childhood social class was predictive of adult obesity. However, adult social class had no influence on adult obesity after adjusting for childhood social class. |
| Hart et al., 2008 [21] | Carstairs deprivation category; paternal occupational social class; adult occupational social class | Obesity (BMI ≥ 30.0 kg/m^2); waist circumference; mean BMI | Renfrew and Paisley | 1,040 male and 1,298 female offspring between 30-59 years old from the Renfrew/Paisley studies | Longitudinal analysis of the relation between childhood and adulthood occupational social class and cardiovascular disease risk factors in adulthood | Age, paternal social class, and adult social class. Models stratified by gender. | Women in manual social classes had higher BMI, larger waists, and were more likely to be obese than women in non-manual social classes. Women with fathers in manual social classes had higher BMI, had larger waists, and were more likely to be obese than women with fathers from non-manual social classes. There were no significant relationships between social class or paternal social class and metrics of obesity among men. In fully adjusted multivariate models, only manual social class was a predictor of higher waist circumference among women. Among men, the downwardly mobile had the highest BMI and waist circumference relative to all other groups. Among women, the stable manual group had the highest rate of obesity. There were no significant differences in obesity risk, mean BMI, or mean waist circumference by mobility. |
| Heraclides et al., 2008 [31] | Paternal occupational social class | Mean BMI; mean waist/hip ratio | London | 3,364 white men and 1,234 white women aged 44-69 who participated in the Whitehall II cohort | A longitudinal analysis of mediators of the relation between father's social class and adult obesity | Age, paternal social class, adult employment grade, educational attainment, diet score, physical activity, alcohol consumption, smoking status. Models stratified by gender. | In multivariate regression models adjusted for adult employment grade, father's social class was inversely associated with BMI and waist/hip ratio in women, but not in men. Adjusting for educational attainment and health behaviors did not attenuate this relationship, although educational attainment explained 10-15% of the effect of father's social class on metrics of obesity. |
| Kuh et al., 2002 [22] | Paternal occupational social class (or household crowding if unavailable); adult occupational social class | Waist/hip ratio; waist circumference | England, Wales, and Scotland | 1,589 men and 1,585 women followed since birth in 1946 to age 43 | A longitudinal analysis of the relations between birth weight, childhood growth, and abdominal obesity in adulthood | Birth weight, relative weight at 7 years, adult BMI, paternal social class, adult social class. Models stratified by gender. | In both unadjusted and adjusted (for birth weight, relative weight at age 7 years, adult BMI, and child and adult social class) regression models among men, both childhood and adult manual social class were associated with higher mean waist/hip ratio and waist circumference compared to non-manual class. Among women, in unadjusted regression models, both childhood and adult manual social class were associated with higher mean waist/hip ratio and waist circumference compared to non-manual class. In models adjusted for birth weight, relative weight at age 7 years, adult BMI, and child and adult social class, only adult manual social class was associated with higher waist/hip ratio and waist circumference. |
| Langenberg et al., 2003 [32] | Paternal occupational social class; head-of-household occupational social class at ages 26 and 43 | Waist/hip ratio; waist/height ratio; body mass index; waist circumference | England, Wales, and Scotland | 1,472 men and 1,563 women followed since birth in 1946 to age 53 | A longitudinal analysis of the relation between lifetime socioeconomic status and central and total obesity in adulthood | Paternal social class, adult social class at age 26, adult social class at age 43. Mobility analyses adjusted for smoking and exercise. Models stratified by gender. | Among men, father's social class was inversely associated with all measures of obesity at age 53. Adult social class at age 26 was inversely associated with all measures of obesity at age 53 except waist circumference. Among women, measures of social class at all ages were inversely associated with all measures of obesity. After adjusting for social class at all ages, there was a significant inverse association between father's social class and all metrics of obesity among men and women, except for waist-hip ratio among women. Among men, adult social class was not associated with any metric of obesity. Among women, adult social class at age 26 was associated with waist/hip ratio, waist/height ratio, and waist circumference. Social class at age 53 was associated with all obesity metrics. There were interactions between sex and social class, indicating that father's social class was more important in determining obesity among women than men. Among those who maintained social class, those who stayed in classes I and II had the lowest levels of obesity. For those who moved up or down in social class, obesity metrics intermediated the levels they left and entered. |
| Lawlor et al., 2005 [33] | Paternal occupational social class at birth; educational attainment; adult occupational social class; annual income | Overweight (BMI ≥ 25 kg/m^2); mean BMI | Aberdeen (as children) | 7,184 individuals born in Aberdeen, Scotland between 1950-1956 | A longitudinal analysis of the relation between childhood socioeconomic status and cardiovascular disease risk factors in adulthood | Age, gender, adult social class, adult income, family size, birth order, birth weight, childhood height, childhood BMI, cognitive function at 7 years, and cognitive function at 11 years, parental smoking, paternal social class, educational attainment | Paternal social class was inversely associated with prevalence of overweight. The prevalence of overweight was 62% among the lowest class group, and 48.5% among the highest group. Mean BMI also increased with decreasing social class. After adjusting for age, gender, adult social class, adult income, family size, birth order, birth weight, childhood height, childhood BMI, cognitive function (at 7 and 11 years), and parental smoking, paternal social class remained associated with 20% higher odds of overweight. After adjusting for educational attainment as well, there was no longer a significant association. |
| Lyratzopoulos et al., 2005 [44] | 1991 enumeration district Townsend Material Deprivation Score | Body mass index > 30 kg/m^2 | Stockport | 11,158 women and 9,831 men aged 35-55 who had a first screening between 1989-1993 and a subsequent screening before 1999 | A longitudinal study of the relation between deprivation and obesity in middle age | Townsend deprivation quintile, age, follow-up time, and baseline BMI. Models stratified by gender | Deprivation was associated with both prevalence of obesity and mean BMI among both men and women. Among men, prevalence of obesity in the least deprived areas was 8.8% compared to 15.7% in the most deprived areas. Among women, 7.8% of women in the least deprived areas and 17.6% in the most deprived areas were obese. There was no significant trend in mean annual BMI increase by deprivation group overall or among those who were not obese. However among those who were obese at baseline, there was a significant association between deprivation and annual increase in BMI. Among women, after adjusting for baseline BMI, there was a significant association between deprivation and annual increase in BMI. |
| Martikainen and Marmot, 1999 [42] | Employment grade | Body mass index; waist/hip ratio | London | 5,507 men and 2,466 women who participated in the first and third phases of the Whitehall II study and were between 35-55 years old during phase I | Longitudinal analysis of the relation between socioeconomic status and weight gain | Employment grade, age. Models stratified by gender | At follow-up, relative to those in grade I (highest), age-adjusted odds of being above the 80th percentile in BMI and waist/hip ratio in grade III among men were 1.39 and 2.51 respectively and among women were 1.72 and 2.21 respectively. Among men, those in grade III had 2.5 times higher odds of experiencing a BMI increase of >6 than those in grade 1. Among women, those odds were 2.8 times higher. |
| Okasha et al., 2003 [34] | Paternal occupational social class | Mean BMI; overweight (BMI ≥ 25 kg/m^2) | Glasgow | 15,322 alumni of the University of Glasgow examined at the University Health Service between 1948 and 1968 | A longitudinal analysis of the relation between childhood socioeconomic status and adult obesity | Childhood social class, age at first examination | Among men, there was a significant association between childhood social class and mean BMI at second (mean 38.9 years) follow-up but not first. There was no association among women. However, there was also an association between childhood social class and risk for overweight at second follow-up among both men and women. There was also a significant trend in mean BMI change between first check-up and second follow-up by social class among both men and women. |
| Pierce and Leon, 2005 [35] | Paternal occupational social class; adult occupational social class | Mean BMI | Aberdeen (as children) | 3,743 Scottish females born between 1950 and 1955 who enrolled in the Aberdeen children of the 1950s study | A longitudinal analysis of the relation between age at menarche and adult BMI | Age at menarche, age, social class at birth, adult social class, parity, smoking, alcohol intake, childhood BMI (as a Z-score within consecutive 3-month bands from 48-83 months), change in BMI per 1-year increase in age at menarche | In bivariate models, both father's social class and adult social class were associated with mean adult BMI. Difference in mean BMI from lowest to highest father's social class was 2.3 kg/m2, while the difference was 1.5 kg/m2 from lowest to highest adult social class. |
| Power et al., 1997 [45] | Paternal occupational social class; educational attainment at age 23; | Overweight (BMI 25.0-29.9 kg/m^2) and obesity (BMI ≥ 30.0 kg/m^2) | England, Wales, and Scotland | 11,407 enrolled in the National Child Development study born in March, 1958 | A longitudinal analysis of changes in social gradients in health metrics between ages 23 and 33 | Adult social class at age 23 or adult social class at age 33 | Among men, the poorest at birth had 2.82 and 1.60 higher odds of overweight at 23 and 33, respectively, and 4.80 and 2.19 higher odds of obesity, respectively compared to the richest. Among women, the poorest at birth had 2.09 and 1.54 higher odds of overweight at 23 and 33, respectively, and 2.84 and 1.99 higher odds of obesity, respectively compared to the richest. There was no change in the gradient of inequality between ages 23 and 33 among either men or women in overweight or obesity based on father's social class. However there was a decrease in the overweight and obesity gradient between ages 23 and 33 among both men and women based on highest attained education. |
| Power and Moynihan, 1988 [37] | Paternal occupational social class; adult occupational social class | Overweight (BMI 25.0-29.9 kg/m^2) and obesity (BMI ≥ 30.0 kg/m^2) | England, Wales, and Scotland | 9,350 young adults aged 23 enrolled in the National Child Development Study born in March, 1958 | A longitudinal analysis of the relation between childhood socioeconomic status and adult overweight | Models stratified by social class and gender | In bivariate analysis, there were significant associations between adult social class and weight distribution, with poorer classes having higher prevalences of obesity and overweight among both men and women. Manual paternal social classes tended toward higher prevalence of obesity at both ages 7 and 23 among males and females. Children from manual households who had higher BMIs at age 7 were more likely to maintain high BMIs (greater than 1.5 standard deviation score) than those from non-manual backgrounds. A greater percentage of children from manual backgrounds compared to non-manual backgrounds became overweight between ages 7 and 23. Among both males and females, among manual groups, high BMI SDS at age 7 was a greater predictor of high BMI SDS score at age 23 than among non-manual groups. |
| Power et al., 2003 [38] | Paternal occupational social class (at age 7, 11, and 15) and adult social class at ages 23 and 33; educational attainment | Obesity (BMI ≥ 30 kg/m^2 at age 33, and defined as above the 90% of the population at other age points) | England, Wales, Scotland | 3,267 men and 3,808 women born in 1958 followed to age 33 | A longitudinal analysis of the relation between childhood socioeconomic status and obesity | Childhood social class, adult social class at age 23, education and parental BMI. Models stratified by gender. | Among men, in cross-sectional analyses through age groups, social class was only associated with obesity at ages 23 and 33. Among women, social class became associated with obesity at ages 16, 23, and 33. Among men, after adjusting for social class at all ages, social class at 7 and 23 predicted obesity at age 33. Among women, only social class at 7 predicted obesity at age 33. In fully adjusted models (including education, class at birth, class at 23 years, and parental BMI), among men, all predictors except class at age 23 were associated with obesity at age 33. Among women, all predictors were associated with obesity at age 33. All associations between social class and obesity were inverse. Prevalence of obesity decreased with educational attainment among both men and women (although no test of significance was applied). |
| Power et al., 2005 [39] | Paternal occupatonal social class; adult occupational social class | Obesity (BMI ≥ 30 kg/m^2) | England, Scotland, Wales | 9,350 men and women aged 33 who were born in one week in 1958; and 3,266 men and women aged 36 who were born in one week in 1946 | A cross-national longitudinal analysis of the relations between childhood socioeconomic status and smoking and obesity | Adult social class, age, childhood social class | Among men, childhood manual social class was associated with higher obesity risk compared to non-manual social class before and after adjusting for adult social class in 1958, but not 1946. Among women, childhood manual social class was associated with higher obesity risk compared to non-manual social class before and after adjusting for adult social class in both years. Among men, adult manual social class was associated with higher obesity risk compared to non-manual social class before and after adjusting for childhood social class in 1958, but there was no relation in unadjusted or adjusted models in 1946. Among women, adult manual social class was associated with higher obesity risk compared to non-manual social class before and after adjusting for childhood social class in both years. |
| Power et al., 2007 [40] | Paternal occupational social class at birth; adult occupational social class | Mean body mass index | England, Scotland, Wales | 9,377 men and women aged 45 who were born in 1 week in 1958 | A longitudinal analysis of the relation between childhood socioeconomic status and cardiovascular disease risk factors | Childhood social class, gender, adult social class | There was an inverse significant relation between childhood social class and BMI even after adjusting for sex and adult social class. There was also an inverse significant relation between adult social class and BMI among both men and women even after adjusting for childhood social class. This relationship was stronger among women than among men. |
| Viner and Cole, 2005 [94] | Paternal occupational social class, maternal education attained; occupational status, annual net income, employment history, educational/vocational attainment | Childhood obesity (BMI > 95%), adult obesity (BMI >28.5 kg/m^2) | England, Scotland, Wales | 8,490 adults born in April 1970 who participated in the 1970 British cohort study. | A longitudinal analysis of the relation between childhood obesity and socioeconomic and psychological outcomes in adulthood | Maternal education, childhood social class, adult social class, maternal and paternal BMI, height at age 10, height at age 30. Models stratified by gender. | In bivariate analyses, among men, those who were obese at childhood and adulthood were significantly more likely to have left school at ≤16 years old and to have left school with no qualifications. Those with childhood and adulthood obesity as well as those with obesity only in childhood were more likely to have unskilled occupational class at adulthood. Among women, those with obesity in adulthood or in childhood and adulthood were significantly more likely to have unskilled occupation. Those who had ever been obese were significantly more likely to have left school at ≤16 years old and with no qualifications, and were more likely to be in unskilled occupational classes. In fully adjusted multivariate models, persistent obesity in men was not significantly associated with any adverse adult outcomes measured, while in women, persistent obesity predicted higher risk of never having been gainfully employed. In men, obesity limited to adulthood was associated with higher risk of leaving school with no qualifications. |
| Wannamethee et al., 1996 [41] | Paternal occupational social class; | Obesity (BMI ≥ 28 kg/m^2) | England, Scotland, Wales | 5,934 men aged 40-59 years at enrollment in the British regional heart study in 1978-1980 followed up in 1992 | A longitudinal analysis of the relation between paternal occupational social class and cardiovascular disease in later life among men | Paternal social class, age, adult social class | In both unadjusted models and those adjusted for age and adult social class, manual paternal social class was associated with higher risk for obesity. In bivariate models stratified by adult social class, among those with non-manual adult social class, manual paternal social class was associated with higher risk for obesity (p<0.01). This association was also seen among those with manual adult social class. |
| **Cross-sectional** |  |  |  |  |  |  |  |
| Baker et al., 1988 [46] | Occupational social class | Body mass index | Caerphilly and Bristol | 2,512 men between 45-59 in Caerphilly and 2,348 men between 45-59 in Bristol | A cross-sectional analysis of the relation between social class and risk factors for ischemic heart disease | N/A | Social class was not associated with body mass index in either Caerphilly or Bristol, although those in higher social classes had a tendency toward lower BMI. |
| Bhopal et al., 2002 [47] | Head-of-household occupational social class; educational attainment; 1991 census enumeration district Townsend deprivation index | Waist circumference and waist/hip ratio | Newcastle | 840 European white, and 709 ethnic Asian men and women between 25-74. | A cross-sectional analysis of the relations between ethnicity, socioeconomic status, and cardiovascular disease risk factors | Men: Social class, social class*ethnicity. Women: education, education*ethnicity. Models stratified by ethnicity and gender. | Among men, Townsend deprivation score significantly predicted waist circumference among Pakistanis. Among European whites, low social class and high Townsend deprivation scores were predictors of high waist/hip ratio. Among Indians and Pakistanis, high Townsend deprivation scores were predictors of waist/hip ratio. There were no associations among Bangladeshis. Among women, social class predicted waist circumference, waist/hip ratio, and BMI among European women, but not among any others. Low education predicted high waist/hip ratio among Indian women. There were no other significant predictors of any metric among any group among women. |
| Brunner et al., 1997 [48] | Salary scale grade | BMI, waist circumference, and waist/hip ratio | London | 4,978 men and 2,035 women aged 39-63 who took part in the Whitehall II study | A cross-sectional analysis of socioeconomic variation in metabolic syndrome | Men: employment grade, age. Women: employment grade, age, menopausal status. Models stratified by gender. | There were significant inverse associations between salary scale pay grade, BMI, waist circumference and waist/hip ratio among both men and women in bivariate models. In multivariate models, low salary scale grade was associated with higher waist/hip ratio in the highest quintile among both men and women. |
| Ellaway et al., 1997 [49] | Manual occupational social class; neighborhood poverty | BMI (overweight 25-29.9 kg/m^2, and obese >30 kg/m^2), waist circumference, waist/hip ratio | West of Scotland | 142 males and 176 females aged 40 years, and 167 males and 206 females aged 60 years who took part in the West of Scotland study | A cross-sectional analysis of the relation between neighborhood surroundings and metrics of obesity in the West of Scotland | Age, gender, social class, smoking status, neighborhood | Social class was significantly associated with BMI distribution with lower social classes having higher prevalence of obesity. The prevalence of obesity among the lowest social class was 27.2% as compared to 20.5% among the highest social class. The poorest neighborhood had an obesity prevalence of 29.2% compared to the richest at 14.2%. Neighborhood was also correlated with BMI and mean waist circumference. After adjusting for all demographic and socioeconomic variables, poorer neighborhood was associated with higher BMI, waist circumference, and waist/hip ratios. |
| Fehily et al., 1984 [50] | Occupational social class | Mean BMI | Caerphilly | 493 men aged 45-59 years | A cross-sectional analysis of the relations between smoking, socioeconomic status, and body mass index | Social class, sucrose intake, protein intake, smoking | There was a significant relationship between social class and mean BMI, and the lowest social class had a mean BMI of 28.2 kg/m2 compared to 26.0 kg/m2 among the highest social class. |
| Gulliford et al., 1992 [51] | Head-of-household occupational social class; educational attainment | Mean BMI; overweight (BMI >25 kg/m^2) | England, Scotland | Parents of 5,229 children who entered the National Study of Health and Growth between 1973-6 and 1982-8 | A serial cross-sectional analysis of obesity prevalence over time | Men: year of study, social class, study area. Women: year of study, age, parity, husband's social class, study area. Models stratified by gender. | Among men, in 1973-6, prevalence of overweight among the lowest occupational group was 36.5% as compared to 30.7% among the highest group. In 1982-8, prevalence of overweight among the lowest occupational group was 40.6% compared to 31.4% among the highest group. Among women, in 1973-6, prevalence of overweight among the lowest occupational group was 24.7% as compared to 13.7% among the highest group. In 1982-8, prevalence of overweight among the lowest occupational group was 28.2% compared to 15.4% among the highest group. Woman's educational attainment was negatively associated with BMI, but there was no longer an association after adjusting for occupational social class. After adjusting for year of study, number of children, and study area, social class was inversely associated with BMI among women. After adjusting for year of study and study area, social class was also significantly inversely associated with BMI among men. |
| Moon et al., 2007 [56] | Ward-level male economic inactivity; ward-level income; ward-level social grade; ward-level proportion renting from the local authority | Overweight (BMI 25.0-29.9 kg/m^2) and obesity (BMI ≥ 30.0 kg/m^2) | England | 18,526 individuals sampled in the Health Survey for England, 1998-1999 | A cross-sectional multilevel analysis of variation in overweight and obesity by locality in England | Sex, age, ethnicity, ward-level % low social grade, ward-level % households with dependent children, ward-level % households renting from the local authority, ward-level % married, ward-level % high social grade | Ward-level percentage with low social grade was associated with higher risk for obesity, but not overweight, while percentage with high social grade was associated with lower risk for overweight, but not obesity. Ward-level percentage renting from the local authority was associated with higher obesity and overweight risk. |
| Parkes, 2003 [52] | Jarman Underprivileged Area Score (based on 1981 Census data); average annual unemployment (1986-1990) | Obesity (BMI ≥ 30.0 kg/m^2) | Rotherham | 3,877 respondents to a mail survey of 22 electoral wards in the Rotherham District Health Authority | A cross-sectional analysis of the relation between area-level deprivation and morbidity | Marital status, educational attainment, work activity level, smoking | There were non-significant positive correlations between Jarman score and obesity, as well as unemployment and obesity. |
| Power et al., 2008 [53] | Adult occupational social class | Obesity (BMI ≥ 30 kg/m^2) | England, Wales, and Scotland | 3,614 men and 3,560 women at age 45 who participated in the National Child Development Study, and were born in March,1958 | A cross-sectional analysis of socioeconomic differences in the clustering of cardiovascular disease risk factors | N/A | Among both men and women, manual social class was associated with significantly higher risk for obesity, with 27.5% and 26.5% obese in the non-manual group among men and women, respectively, and 28.0% and 27.5% obese among men and women in the manual group, respectively. |
| Riva et al., 2009 [59] | Adult occupational social class; access to a car; years of residence in local area; area-level deprivation | Overweight (BMI ≥ 25 kg/m^2) | England | 30,776 respondents aged 18 years older who participated in the Heath Survey for England, 2000-2003 | A cross-sectional analysis of urban-rural disparities in health, as well as socioeconomic inequalities in health in rural areas | Gender, age, ethnicity, occupational social class, access to a car, residential stability. Models stratified by region (London, other cities, semi-rural areas, villages) | Area-level deprivation across all of England was associated with higher obesity odds in models adjusted for individual characteristics and urbanicity. In models adjusted for other individual risk factors, in London, only semi-skilled manual workers had higher risk than professionals of overweight, in other cities and semi-rural areas, social class was associated with odds of overweight. There was no association in villages. Access to a car was only associated with odds of overweight in other cities (not London). Years of residence in local areas were not associated with odds of overweight in any context. In other cities, and semi-rural villages, higher area-level deprivation was associated with risk for overweight. |
| Rona and Morris, 1982 [54] | Head-of-household occupational social class; head-of-household employment status | Weight/height | England, Scotland | 7,256 married mothers and fathers of children aged 5-11, themselves aged 20-55 in 1972 | A cross-sectional analysis of the relation between social factors and obesity among adults in Scotland and England | Household social class, number of children, Head-of-household employment status, age, spouse's weight/height. Models stratified by country and gender. | Among males, in unadjusted analysis of variance, social class was associated with weight/height in both England and Scotland. This was true among women in both contexts, as well. After adjusting for number of children, age, male employment status and spouse’s weight/height, social class remained associated with weight/height among men in England and women in both contexts. It was marginally associated (p<0.1) with weight/height among Scottish men. Employment status was predictive of weight/height in unadjusted models among men in Scotland and women in both contexts, and in adjusted models among men and women in England and men in Scotland. It was marginally associated with weight/height among women in Scotland. There was an interaction between age and social class among men. In England, unemployed men under 35 had higher risk for weight/height greater than the 90%, while among Scottish men, unemployed men older than 45 had risk for weight/height greater than the 90%. Among both males and females, head-of-household social class was associated with risk for weight/height greater than the 80th and 95th percentile (p<0.1), with relative risk greater among men than among women. |
| Scarborough and Allender, 2008 [55] | Head-of-household occupational social class | Overweight (BMI 25.0-29.9 kg/m^2) and obesity (BMI ≥ 30.0 kg/m^2) | England | Respondents in the 1993-2004 Health Surveys for England aged 16 and above | A serial cross-sectional analysis of changes in geographic disparities in overweight and obesity between the North and South of England | Age, social class. Models stratified by gender. | In models adjusted for geographic location and age, social class was a significant predictor of obesity among both genders in all age groups in all time periods. In similar models of overweight, social class was a significant predictor of overweight among women, but not men in any age group in any time period. |
| Wardle et al., 2002 [57] | Age at leaving full-time education; head-of-household occupational status; government financial aid receipts; owned accommodation | Obesity (BMI ≥ 30 kg/m^2) | England | 15,061 individuals sampled in the Health Survey for England, 1996 | A cross-sectional analysis of the relation between socioeconomic status and obesity | Age at leaving education, occupational social class, receipt of benefits, housing status, age, ethnicity, marital status. Models stratified by gender. | Among men, those in the skilled manual class had the highest prevalence of obesity (19.2%), while among women, unskilled manual had the highest prevalence of obesity (27.4%). In bivariate and multivariate analyses (including education, occupational status, benefits, housing, age, marital status, and ethnicity), earlier age of leaving school was associated with higher obesity odds among both men and women. Lower occupational class was associated with higher obesity odds among both men and women in bivariate models, but only women in multivariate models. Receipt of benefits was associated with higher obesity odds among both men and women in both adjusted and unadjusted models. Renting was associated with higher obesity odds in women in both bivariate and multivariate models, but not among men. |
| Wardle and Boniface, 2008 [95] | Occupational social class; educational attainment | Mean BMI, obesity (BMI >30 kg/m^2), BMI >35 kg/m^2, BMI >40 kg/m^2, mean waist circumference | England | 20,246 individuals sampled in the Health Survey for England, 1993-1994 and 11,708 individuals sampled in the Health Survey for England, 2002-2003 | A serial cross-sectional analysis of changes in the social distribution of body mass index between 1993 and 2003 | N/A | There was no difference in increase in mean BMI, proportion obese, proportion with BMI>35 kg/m2 or proportion with BMI>40 kg/m2 between 1993-2003 by social class or educational attainment. |
| Weatherall and Shaper, 1988 [58] | Occupational social class; proportion of manual workers | Mean BMI; obesity (BMI ≥ 28.0 kg/m^2) | England, Wales, and Scotland | 7,735 men aged 40-59 who took part in the British Regional Heart Study | A cross-sectional analysis of the correlates of obesity | Mean BMI by social class stratified by smoking status | The richest social class had the lowest mean BMI and the lowest proportion of obese men. Social class III-manual had the highest mean BMI and the highest proportion of obese men. While all other groups had higher mean BMI than social class I, only social class II and social class III-M were significantly higher. By town, there was a significant correlation between the proportion of manual workers in a town and the proportion obese in that town. |

*Includes independent covariates included in final models assessing the relation between metrics of socioeconomic position and obesity
